# Twenty-one new sequence markers for population genetics, species delimitation and phylogenetics in wall lizards (*Podarcis* spp.)

**DOI:** 10.1186/1756-0500-6-299

**Published:** 2013-07-27

**Authors:** Carolina Pereira, Alvarina Couto, Carla Luís, Diogo Costa, Sofia Mourão, Catarina Pinho

**Affiliations:** 1 CIBIO/UP, Centro de Investigação em Biodiversidade e Recursos Genéticos, Universidade do Porto, Campus Agrário de Vairão, Rua Padre Armando Quintas, 4485-661 Vairão, Portugal; 2 Faculdade de Ciências da Universidade do Porto, Rua do Campo Alegre, s/n, 4169-007 Porto Portugal

**Keywords:** *Podarcis*, Polymorphism, Lizards, Squamata, Lacertidae, Nuclear sequence marker, Genetic diversity

## Abstract

**Background:**

Wall lizards of genus *Podarcis* are abundant and conspicuous reptiles inhabiting Europe and North Africa. In recent years, they have become a popular lizard model for phylogeographical and evolutionary ecology studies. However a lack of suitable nuclear markers currently presents a limitation on analyses of molecular evolution within this genus. We address this limitation by developing twenty-one new primer pairs for polymerase chain reaction (PCR) amplification and sequencing of anonymous sequence markers in *Podarcis vaucheri* and performed an assay of their cross-amplification and polymorphism levels in two closely- (*P. bocagei* and *P. liolepis*) and two distantly-related (*P. muralis* and *P. tiliguerta*) congeners.

**Findings:**

Cross-amplification and sequencing was straightforward among members of the Iberian and North-African group within genus *Podarcis* (which includes *P. vaucheri*), and somewhat less successful in species belonging to other groups (one and four loci out of 21 failed to amplify in *P. muralis* and *P. tiliguerta*, respectively, and overall success rates were lower). Nucleotide diversity for the five species examined ranged from 0.35% to 3.5%, with an average of 1.5% across all loci. Insertion and deletion polymorphisms were found in all but three loci.

**Conclusions:**

Given the high cross-amplification rates, these markers constitute a valuable addition to set of genomic resources available for *Podarcis*, especially in studies dealing with phylogenetics, species delimitation, population genetics and phylogeography.

## Findings

Wall lizards of the genus *Podarcis* (Squamata: Lacertidae) are among the most conspicuous, abundant and widely distributed reptiles in Europe and North Africa. The genus currently comprises 21 fully recognized (but likely many more) species and exhibits a circum-mediterranean distribution. A recent review [1] indicated that *Podarcis,* more than any other genus of lizard including *Anolis*, has been used extensively in studies pertaining to evolutionary ecology and phylogeography.

Nevertheless, there is a striking paucity of genetic markers available for studying the evolution and ecology of this genus. Apart from allozymes, which are impractical for high throughput projects [2], mitochondrial DNA, microsatellite loci, which show extensive levels of homoplasy between species e.g. [3,4] and a few introns [5,6], the genetics tool kit for the genus is very limited, especially in terms of nuclear markers. This is particularly problematic given that the taxonomy of the genus is highly unstable and both inter and intraspecific diversity remain poorly understood. Species such as *P. erhardii*[7], *P. hispanica*[8] and *P. tiliguerta*[9] have been suggested to be cryptic species complexes, with non-negligible gene flow among species and pervasive incomplete lineage sorting in nuclear genes [5]. Scenarios of extensive mitochondrial DNA introgression among species have furthermore been reported [10], implying that mtDNA markers alone should not be used for barcoding purposes in this genus. These features imply that a correct assessment of species boundaries and speciation dynamics require more detailed genetic studies based on a large battery of different types of genetic markers.

This paper describes primers for 21 nuclear loci in the Moroccan wall lizard *Podarcis vaucheri* as well as their utility in four other congeners (*P. bocagei, P. liolepis, P. muralis* and *P. tiliguerta*). *P. vaucheri* is one of the most widespread and genetically structured species within the clade [8,11]. It is also the only species in the genus found on both sides of the Strait of Gibraltar, a feature that makes this species a valuable biogeographic model.

Total genomic DNA from a single individual of *P. vaucheri* (Bab Taza, Chefchaouen province, Morocco) was digested with TasI (Fermentas, Vilnius, Lithuania) according to the manufacturer’s protocol.

The digested genomic DNA was subsequently ligated to TSPADSHORT and TSPADLONG adapters [12]. In order to do so, TSPADSHORT and TSPADLONG were previously ligated to each other to obtain a double-strand adapter by adding 89.2 μL of Tris-EDTA buffer, 0.8 μL of NaCl 5 M, and 5 μL of each oligonucleotide (100 μM). The mixture was placed at 95°C for 3 minutes, 65°C for 2 minutes, 45°C for 2 minutes and 25°C for one minute. The double-stranded adapter was then ligated to the digested genomic DNA by adding 50 μL of digestion with 10 μL of the TSPADSHORT/TSPADLONG adapter, 36 units of T4 DNA ligase (Promega, Fitchburg, Winsconsin, USA) and 28 μL of reaction buffer (300 mM Tris–HCl, pH 7.8, 100 mM MgCl_2_, 100 mM DTT and 10 mM ATP), followed by an incubation at 16°C overnight.

Subsequently, the DNA linked to the double-stranded adapter was used as template for polymerase chain reaction (PCR) using TSPADSHORT as primer. Five replicate 25 μL reactions were carried out, each containing 1X PCR buffer (50 mM Tris–HCl, 50 mM NaCl, pH 8.5); 2 mM MgCl_2_; 1 mM each dNTP, 2U of GoTaq DNA polymerase (Promega), 1 μM of TSPADSHORT and 1,25 μL of the DNA-adapter mixture. Amplification conditions consisted of a step at 72°C for 5 minutes, followed by 25 cycles with 1 minute at 94°C, 1 minute at 55°C and 1 minute at 72°C.

A mixture of the five PCR replicates was then ran on an agarose-TBE gel and fragments of ~400–2000 bp were gel-isolated using the GE Healthcare Life Sciences (Uppsala, Sweden) Illustra GFX PCR DNA and Gel Band Purification kit. After purification, PCR products were ligated into pGEM-T Easy Vector Systems kit (Promega) according to the manufacturer’s instructions. The output of the ligation reaction was then transformed into *Escherichia coli* competent cells and grown on standard LB medium with ampicillin/IPTG/X-Gal (details on this medium can be found on the pGEM-T Easy Vector Systems protocol); conventional blue/white screening was used to discriminate clones that contained inserts (white) from those that did not (blue).

Using this protocol, a total of 72 positive colonies were obtained. These samples were then amplified using universal primers pUC/M13F and pUC/M13R. PCR reactions were carried out in 25 μL volumes containing 1X PCR buffer, MgCl_2_; 1 mM each dNTP, 2U of GoTaq DNA polymerase (Promega), 0.4 μM each primer and 2 μL of colony DNA. After verification of successful amplification, the inserts were sequenced from both strands with the same primers used for amplification. Sequencing was performed on an Applied Biosystems 3130xl Genetic Analyzer (Applied Biosystems, Foster City, California).

Sequences were subject to a previous pruning step in order to remove vector sequences. Subsequently, in order to prevent designing primers in repetitive regions, which would lead to difficulties in amplification and/or to the amplification of paralogs, we discarded clones containing putative repetitive elements. In order to identify such clones, we performed a BLAST search [13] against the NCBI (http://www.ncbi.nlm.nih.gov/) nucleotide database and a RepeatMasker [14] run. We also used the standalone version of BLAST to remove putative duplicates and sequences comprising potential repetitive elements not identified by the previous methods. Clones that were excessively small (i.e. with an insert size below 300 bp) were also discarded.

Thirty-three clone sequences passed this initial quality-control step and were therefore used as template for primer design. These primers were preliminarily tested and optimized by screening across a set of 16 individuals from *P. vaucheri* and other species. Amplifications were carried out in a similar fashion for all loci: in 25 μL volumes, containing 1X PCR buffer; 3 mM MgCl_2_; 0.6 mM each dNTP, 2U of GoTaq DNA polymerase (Promega), 0.4 μM each primer and approximately 50 ng of genomic DNA. Amplification conditions consisted of a denaturation step at 92°C (3 minutes), followed by 40 cycles of denaturation at 92°C (30 seconds), an annealing step of 30 seconds and extension at 72°C for 1 minute. The first 25 of these cycles were conducted in a touchdown manner, with annealing temperature decreasing 0.5°C per cycle from 62°C to 50°C, and in the last 15 cycles the annealing temperature was constant at 50°C. A final extension was carried out at 72°C for 15 minutes. Sequencing was performed in a Applied Biosystems 3130xl Genetic Analyzer.

According to this preliminary screening, 23 primer pairs showed consistent amplification and sequencing success. Two of these presented some evidence for the amplification of paralogs (such as the presence of the same heterozygous positions in all sequences) and were further excluded from the analyses.

**Figure 1:**
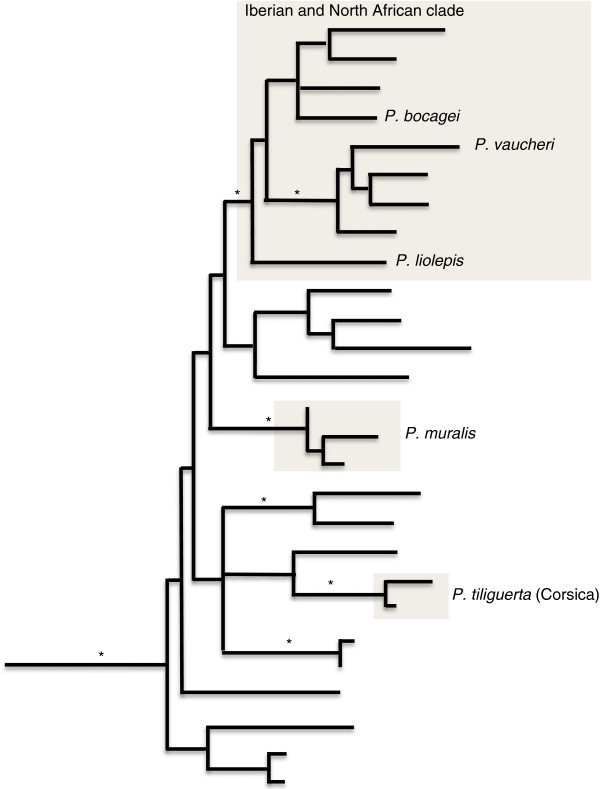
**Schematic representation of the current hypothesis for evolutionary relationships within *Podarcis *(according to**[9]**, based strictly on mitochondrial DNA), with an emphasis on the species included in this study.** Please note that *P. tiliguerta* appears to be a complex of species; in this study only the Corsican form (highlighted in the figure) was included. Clades marked with an asterisk are statistically well-supported.

Primer sequences for the remaining 21 loci are reported in Table 1. These loci were chosen for a more detailed examination of variation in 49 individuals, including 13 *P. vaucheri*, 12 *P. liolepis*, 10 *P. bocagei*, nine *P. muralis* and five Corsican *P. tiliguerta* (see Additional file 1 for a complete list of the individuals used and their sampling localities). The first three species belong to the Iberian and North African clade, hence are closely related in comparison to the other two species, which are more distantly related (to the Iberian clade and to each other; see Figure 1 for a schematic representation of the phylogenetic relationships amongst these species based on [9]). Purification and sequencing of PCR products for this panel of samples (amplified using the same conditions described above) were carried out by Macrogen (http://dna.macrogen.com/eng/; Seoul, Korea).

**Table 1:** Sequences of the primers developed in this study

**Locus name**	**Primer**	**Sequence (5′ - 3′)**
*Pod6b*	pod6bf	ctggtaatggcccgctatgtatggg
	pod6br	ataaagctgggaagctcttgagtcc
*Pod7b*	pod7bf	gtcactttggtgctgctcgcacagc
	pod7br	tgtaatgctgcaacttggcgacacc
*Pod11*	pod11f	gactttgggttcaaatctccacccc
	pod11r	aggtcatctgcttgactgttctggc
*Pod12b*	pod12bf	accttcttttgcctacgcacgccag
	pod12br	ctgtccacaacacccttattctgcc
*Pod13*	pod13f	gcagttgttgctgggctcatttctg
	pod13r	acatgattttgaggggacgcaaacc
*Pod14*	pod14f	gctttcctatgaggctcaagtttgg
	pod14r	agccgactgtctctaataacttccc
*Pod14b*	pod14bf	ctggaggaagggtagcatgatctcc
	pod14br	ctgacagccgcatcagacgttcagc
*Pod15*	pod15f	actttacatcccatgataggtctgg
	pod15r	tgatatagcagaacacctgtgcagc
*Pod15b*	pod15bf	aatcctggctaaatgcaagccttgg
	pod15br	gccaggagaataagctactccatcc
*Pod16*	pod16f	ttcctttgttacaccttgggaggggt
	pod16r	ctggagagggagcagcggcttcagg
*Pod17*	pod17f	taattgcccattcccttcgattccc
	pod17r	tgataaccattgccttcattatgcc
*Pod20*	pod20f	gagtgcttacaggctgtgaagatgt
	pod20r	atgccgattcaaccaaaacatggcg
*Pod21*	pod21f	tctagagaccgagtccttgtaaggg
	pod21r	gaaactcctctcccagagaacgacc
*Pod25*	pod25f	gtattatcaggcccagtgcttgtgg
	pod25r	tggtggattatctatcatctgctcc
*Pod31*	pod31f	aacggctatttgcggactacagtag
	pod31r	gcaggtcactaggaatatagaagcc
*Pod33*	pod33f	atctgatgggagagcattccacagg
	pod33r	gtgcgccatattacacagcaactgg
*Pod38*	pod38f	agcgctgcaactttctctgcttccg
	pod38r	gggcatgagtcaggagtagtcacgc
*Pod43*	pod43f	ccattacgtcaagtattgctaatgc
	pod43r	catagagattcttatgcagaactgg
*Pod55*	pod55f	ggatctttataggagagtgcaggcc
	pod55r	ttccagattgtgtttatcctggtgg
*Pod69*	pod69f	ttataagtgtgggagtagcgagctg
	pod69r	ggagcattgaaaatatccaagatgg
*Pod72*	pod72f	gaagggagacggtgtgctattgtcg
	pod72r	cctcctgctctctcttcctaacacg

Sequences were aligned using Sequencher v 4.1.4 [15]. The number of successful sequences obtained for each species was recorded. In sequences that were heterozygous for insertion/deletion (indel) polymorphisms, we used the method outlined by Flot et al. [16] to recover haplotypes. These were subsequently used in PHASE v. 2.1 [17] to assist haplotype reconstruction. PHASE was ran three times per dataset based on 100 burn-in and 100 post burn-in iterations and a thinning interval of 1. We used the minimum value of 0.5 as the posterior probability threshold to accept a given reconstruction, as long as there was consistency among runs. Positions that were resolved inconsistently among runs were considered as “unphased” and were therefore replaced by a “N” in the alignments. Phased alignments were imported into DNAsp [18] for polymorphism analyses. For each gene and species, we calculated nucleotide diversity (π), number of segregating sites (S), number of haplotypes (H) and haplotype diversity (Hd), as well as Hudson and Kaplan’s [19] Rm, indicating the minimum number of recombination events. We also computed the number of indel polymorphisms for each data set. To evaluate the possibility of a close genomic relationship between pairs of our markers, we further performed an exact test for genotypic disequilibrium per species using the program Genepop version 4.1.4 [20].

We obtained a total of 813 individual sequences among the 21 loci analysed in this study. Overall sequencing success results and polymorphism levels are shown in Table 2; detailed information concerning each species is reported in Additional file 2. Between 26 (*Pod25*) and 44 (*Pod55*, *Pod69*) individuals per locus were amplified and sequenced successfully. *P. bocagei* (rather than *P. vaucheri*, the original source of our library)*,* showed the highest sequencing success rate on average (86%), although this value is not significantly different from that in *P. vaucheri* (84%) or *P. liolepis* (83%) (p > 0.05, based on a permutation test). In contrast, cross-amplification in *P. muralis* and *P. tiliguerta* was significantly less successful (71% and 53% success rate respectively) than in any of the Iberian and North African forms (p < 0.05 for all comparisons involving *P. muralis* and p < 0.01 for all comparisons involving *P. tiliguerta*); these values account for the fact that one (*Pod12b*) and four (*Pod6b, Pod13, Pod14, Pod38*) loci failed to amplify or sequence in *P. muralis* and *P. tiliguerta*, respectively. This is in line with the current knowledge on these species evolutionary relationships, since *P. bocagei, P. liolepis* and *P. vaucheri* are all members of the Iberian and North African clade within the genus, whereas *P. muralis* and *P. tiliguerta* are more distantly related [9]. All loci except *Pod43, Pod55* and *Pod69* exhibited indel polymorphisms. With respect to polymorphism levels, for the complete five-species dataset, nucleotide diversity was on average 1.5%, ranging from 0.035% in *Pod43* to 3.5% in *Pod16*. Diversity levels varied extensively among species (see Additional file 2); however, given the limited number of individuals included in this study and the fact that patterns of genetic variation are typically complex, our sampling schemes are not totally comparable across species; intraspecific polymorphism values should therefore be regarded as mere indications, and accordingly we have abstained from interpreting differences in polymorphism levels across species. There was no evidence for linkage disequilibrium between any pair of loci in any species (after Bonferroni correction).

**Table 2:** Summary statistics for the loci developed in this study

**Locus**	**Species sequenced**	**Success rate (%)**	**N**	**Sites (bp)**	**NetSites**	**S**	**H**	**Hd**	**π**	**Rm**	**Indels (size)**	**GenBank accession numbers**
*Pod6b*	PB, PL, PV, PM	76	74	481-491	404	55	33	0.945	0.012	4	6 (7/1/1/5/8/2)	KC680863 – KC680907
*Pod7b*	PB, PL, PV, PM, PT	88	86	379-418	312	42	26	0.918	0.018	2	5 (1/7/20/19/6)	KC680908 – KC680951
*Pod11*	PB, PL, PV, PM, PT	86	84	432-437	344	43	34	0.944	0.016	7	5 (1/M/5/1/1)	KC680952 – KC681005
*Pod12b*	PB, PL, PV, PT	69	68	404-656	288	65	32	0.933	0.025	4	9 (6/7/1/1/1/245/3/3/1)	KC681006 – KC681046
*Pod13*	PB, PL, PV, PM	71	70	367-381	261	33	20	0.713	0.011	3	4 (14/4/1/1)	KC681047 – KC681087
*Pod14*	PB, PL, PV, PM	82	80	459-486	363	33	27	0.918	0.016	4	6 (10/22/21/1/3/9)	KC681088 – KC681136
*Pod14b*	PB, PL, PV, PM, PT	84	82	486-508	348	54	31	0.893	0.016	5	7 (3/M/4/M/28/1/4)	KC681137 – KC681183
*Pod15*	PB, PL, PV, PM, PT	82	80	498-540	370	51	35	0.955	0.021	5	5 (11/2/2/2/1)	KC681184 – KC681227
*Pod15b*	PB, PL, PV, PM, PT	86	84	420-433	399	41	35	0.939	0.009	5	5 (M/13/2/2/M)	KC681228 – KC681284
*Pod16*	PB, PL, PV, PM, PT	76	74	313-364	213	46	38	0.972	0.035	6	11 (18/M/1/5/18/11/7/13/2/11/33)	KC681285 -- KC681332
*Pod17*	PB, PL, PV, PM, PT	88	86	380-405	246	32	29	0.927	0.014	2	7 (14/1/1/25/M/1/1)	KC681333 - KC681399
*Pod20*	PB, PL, PV, PM, PT	82	80	396-410	305	34	27	0.913	0.012	1	4 (1/1/M/13)	KC681400 - KC681448
*Pod21*	PB, PL, PV, PM, PT	86	84	303-316	212	39	24	0.916	0.017	2	4 (9/7/1/13)	KC681449 - KC681492
*Pod25*	PB, PL, PV, PM, PT	53	52	266-294	233	29	21	0.937	0.020	2	4 (1/18/8/2)	KC681493 - KC681522
*Pod31*	PB, PL, PV, PM, PT	69	68	491-909	383	37	28	0.904	0.010	4	13 (1/5/17/1/9/382/10/2/5/10/2/12/2)	KC681523 - KC681565
*Pod33*	PB, PL, PV, PM, PT	86	84	325-340	242	29	30	0.840	0.010	3	4 (1/16/3/M)	KC681566 - KC681614
*Pod38*	PB, PL, PV, PM	80	78	536-539	406	39	15	0.860	0.024	3	5 (1/2/1/2/1)	KC681615 - KC681656
*Pod43*	PB, PL, PV, PM, PT	73	72	394	366	16	17	0.720	0.004	0	0	KC681657 - KC681692
*Pod55*	PB, PL, PV, PM, PT	90	88	423	354	33	26	0.737	0.009	5	0	KC681693 - KC681736
*Pod69*	PB, PL, PV, PM, PT	90	88	388	339	15	15	0.812	0.004	0	0	KC681737 - KC681780
*Pod72*	PB, PL, PV, PM, PT	61	60	469-485	358	52	20	0.884	0.023	8	6 (1/5/11/1/7/14)	KC681781 - KC681810

Species sequenced: *PB Podarcis bocagei, PL P. liolepis*, *PV P. vaucheri, PM P. muralis*, *PT P. tiliguerta. N* number of sequenced gene copies, *Sites* size range of sequences in the alignment, *NetSites* number of sites effectively used for polymorphism calculations (removing indels and Ns), *S* number of segregating sites; *π* nucleotide diversity, *H* number of haplotypes, *Hd* haplotype diversity, *Rm* minimum number of recombination events [15], *Indels* number of insertion/deletion polymorphisms. The size of indels (with respect to each alignment) is reported in parentheses. M means microsatellite-type indel. For the same information detailed by species, please see Additional file 2.

For an additional characterization of these 21 loci, we performed a BLAST search of the sequenced clone sequence against the *Anolis carolinensis* genome (available in the NCBI website). Because *Podarcis* and *Anolis* are very distantly related, we used the “discontinuous megablast” algorithm to perform this search. Only 10 out of the 21 markers show a significant match to the *Anolis* genome (see Additional file 3). In many of these cases, functional annotation of the specific regions of the *Anolis* genome is still incomplete, which prevents an assessment of the putative genomic location of the markers. Only *Pod31* and *Pod33* appear to map within a protein-coding gene. Both loci comprise putatively exonic and intronic sequences: the first ~170 bp of the *Pod31* and the last 86 bp of the *Pod33* alignments are probably exonic, whereas the remaining sequence of both markers is likely intronic. *Pod20, Pod21* and *Pod43* all map to intergenic regions. It is noteworthy that both *Pod20* and *Pod31* map to the *Anolis* chromosome 5. A more detailed look into these markers’ genomic location shows that they are located about ~8 Mb apart in the genome, which could indicate that they may be under at least some physical linkage in *Anolis*. However, we have no evidence for any linkage disequilibrium between this (or any other) pair of loci in *Podarcis*, indicating that the markers may be on a different genomic location in this genus or that recombination rate between the two loci is high enough for the markers to behave as unlinked.

A potential problem in the development of anonymous genetic markers is the possibility of amplification of paralogs instead of single-copy loci. We cannot be completely sure that these markers all correspond to single-copy markers and prospective users of these primers should be aware of this caveat. However, we did our best to minimize this problem by avoiding designing primers in repetitive elements (which are typically found in high copy numbers in the genome) and deliberately excluding loci presenting evidence for the amplification of paralogs. Furthermore, BLAST analyses against the *Anolis carolinensis* genome showed that most loci that did show significant similarity had a single match, which would not be expected if the markers were found in multiple copies. Furthermore, in the four loci that did have more than one significant match (*Pod7b*, *Pod21*, *Pod33, Pod55*) the second most significant hit had a comparatively low total score, indicating that only a few base pairs – and not the whole locus - were found in common. This suggests either spurious matches or perhaps the presence of previously undescribed repetitive elements, but is not an indication of the amplification of paralogs. Finally, we have no evidence for the presence of more than two alleles per locus in any of the individuals sequenced in this study.

Given their high cross-amplification rate and of the existence of substantial intraspecific polymorphism, we predict these markers will be useful both in macroevolutionary (for e.g. species delimitation or phylogenetics) and microevolutionary (population genetics and phylogeography) frameworks. They may also be an important source from which to develop single nucleotide polymorphism (SNP) markers for the genus. Furthermore, albeit we were not able to perform a formal cross-amplification assay on other species, data from our laboratory suggest that a large proportion of the markers amplify well in other lacertid genera such as *Timon* or *Scelarcis* (C. Luís, unpublished data), and that at least *Pod15b* can be easily amplified in *Archaeolacerta bedriagae* (D. Salvi, pers. comm). The newly reported primer pairs are thus a valuable addition to the current genetics toolkit for *Podarcis* and probably for other lacertid species as well.

## Availability of supporting data

The data sets supporting the results of this article are available in the GanBank repository, accession numbers KC680863-KC681810. Files containing all sampling localities, polymorphism and sequencing success values discriminated by species and results from a BLAST analysis against the *Anolis carolinensis* genome are provided as supplemental material.

## Competing interests

The authors declare that they have no competing interests.

## Authors’ contributions

CPereira participated in the study conception, carried out most laboratory work, participated in sequence alignment, in data analyses and in the manuscript preparation. AC, CL and DC participated in sequence alignment. SM participated in laboratory work. CPinho participated in the study conception, carried out laboratory work, participated in sequence alignment and in data analyses and wrote the initial draft of the paper. All authors read and approved the final manuscript.

## Supplementary Material

Additional file 1 Sampling locations for the 49 individuals included in the polymorphism assay. Click here for file

Additional file 2 Summary statistics for the loci developed in this study, detailed by species. Click here for file

Additional file 3 **Results of BLAST analyses of the 21 loci developed in this study against the *Anolis carolinensis *genome.** Click here for file
